# Volatile organic compound profiling as a potential biomarker in irritable bowel syndrome: A feasibility study

**DOI:** 10.3389/fmed.2022.960000

**Published:** 2022-08-04

**Authors:** Kathleen Van Malderen, Nikita Hanning, Helen Lambrechts, Tine Haverhals, Silke Van Marcke, Hannah Ceuleers, Joris G. De Man, Benedicte Y. De Winter, Kevin Lamote, Heiko U. De Schepper

**Affiliations:** ^1^Laboratory of Experimental Medicine and Pediatrics, Faculty of Medicine and Health Sciences, Infla-Med Centre of Excellence, University of Antwerp, Antwerp, Belgium; ^2^Department of Gastroenterology and Hepatology, Antwerp University Hospital, Edegem, Belgium; ^3^Medical School, Faculty of Medicine and Health Sciences, University of Antwerp, Antwerp, Belgium; ^4^Department of Internal Medicine and Pediatrics, Ghent University, Ghent, Belgium

**Keywords:** irritable bowel syndrome, IBS, VOC, volatile organic compounds, ion mobility spectrometry, neurogastroenterology

## Abstract

**Background:**

Irritable bowel syndrome (IBS) is a chronic gastrointestinal disorder for which no diagnostic tools are currently available. Patients are diagnosed using the Rome IV criteria and subtyped into a diarrhea, constipation, or mixed phenotype based on their dominant stool pattern. A recent development in the biomarker area is the analysis of volatile organic compounds (VOCs). The aim of this study was to evaluate the potential of VOCs as diagnostic and phenotypic biomarkers for IBS in breath and fecal samples.

**Materials and methods:**

Breath and fecal samples from IBS patients and healthy asymptomatic controls (HC) were analyzed with multicapillary column/ion mobility spectrometry (MCC/IMS) and classification models were created based upon VOCs and clinical characteristics.

**Discussion:**

Irritable bowel syndrome patients were differentiated from HC by means of volatile profiling in both breath and fecal samples with area under the curve (AUCs) of respectively 0.62 and 0.80. Patient subtypes could also be differentiated from each other with AUCs ranging between 0.65 and 0.78. Furthermore, VOC models could differentiate IBS patients based on clinical characteristics like psychological comorbidities and microbiota-influencing therapies.

**Conclusion:**

This study is the first to demonstrate the use of VOC profiling with the help of MCC/IMS to differentiate IBS patients. Furthermore, the importance of clinical characteristics beside the dominant stool pattern in the differentiation of IBS patients was emphasized.

## Introduction

Irritable bowel syndrome (IBS) is a chronic disorder characterized by abdominal pain and an altered bowel habit. It affects 4–11% of the population making it one of the most prevalent gastrointestinal disorders (1, 2). Four IBS subtypes are described according to the dominant stool pattern: diarrhea (IBS-D), constipation (IBS-C), mixed (IBS-M), and unspecified (IBS-U). IBS has a major impact on the quality of life and is associated with high healthcare costs because of difficulties in diagnosis and treatment (3, 4).

The etiology of IBS is largely unknown, although patients often report an infectious, stressful, or traumatic life event preceding the onset of symptoms. The pathogenesis is multifactorial, and involves increased intestinal permeability, dysmotility, intestinal dysbiosis, food hypersensitivity, visceral hypersensitivity, brain-gut axis dysregulation, inflammation, genetics, and psychological stress (5, 6).

Since there is a lack of biomarkers to aid in identification and follow-up of IBS patients, diagnosis of IBS is currently based on the Rome IV criteria and some tests to exclude specific organic diseases such as celiac disease, inflammatory bowel disease (IBD), or colon cancer (7, 8). A rapidly growing development in the biomarker area is the study of volatomics, focusing on the analysis of volatile organic compounds (VOCs). VOCs are metabolites produced *in vivo* during physiological processes and pathophysiological metabolic activity and are characterized by a low molecular weight (<300 Da) and a high vapor pressure at room temperature (9). Additionally, they can originate from the microbial metabolism, and by metabolization of exogenous products like food or drugs (10). VOCs are excreted in urine, sweat, blood, feces, and exhaled breath, making them easily accessible to study. Since IBS is associated with low-grade inflammation and dysbiosis, volatomics may offer a non-invasive tool to reflect these pathophysiological mechanisms, aiding in diagnosis, treatment, and follow-up (10).

In the past, researchers have tried to differentiate IBS patients from healthy controls (HCs). These studies were mainly based upon VOCs analyzed by gas chromatography–mass spectrometry (GC-MS) and two studies used high field asymmetric waveform ion mobility spectrometry (FAIMS) (10–19). Almost all these studies investigated VOCs in fecal samples with only two looking at VOCs in breath, and two looking at VOCs in urine. Each of these matrices have their own advantages and disadvantages. On the one hand fecal samples are easy to collect and store and might provide a more integral view on gastrointestinal diseases. However, patients are often reluctant to collect and hand in a fecal sample. On the other hand, while breath is easy to collect and use for real-time analysis, there are difficulties in storing samples for later analysis (20). Results of these studies were not consistently positive and resulted in area under the curve (AUCs) ranging between 0.44 and 0.95 (10–19, 21). Sagar et al. (19) looked at patients with bile acid diarrhea and IBS-D and found evidence that metabolic processes of the microbiota are linked to specific VOCs.

At the moment, GC-MS is considered as the golden standard in VOC detection and analysis. It is a highly sensitive technique that allows explicit identification of individual compounds. However, it is also a labor-intensive technique needing trained technicians and associated with high analytical costs. The initial purchase costs for GC-MS are around €250,000 with an approximate cost of €120 per sample afterward. It requires *offline* sampling including different pre-concentration steps which offers the possibility to store samples for batch analysis but also increases the risk of introducing contamination and bias (10, 20, 21). Al these factors limit the use of GC-MS in clinical practice. In this study we examined the possibilities of multicapillary column/ion mobility spectrometry (MCC/IMS). It is an easy to use and less costly alternative compared to GC-MS. The initial purchase costs for MCC/IMS are around €50,000 with an approximate cost of €25 per sample afterward. It provides real-time (*online*) analysis, ameliorating its use in a clinical setting. A potential limitation of MCC/IMS is that it only allows for a (pseudo)identification of compounds making it impossible to identify specific individual compounds (10, 20). For this reason VOCs detected with MCC/IMS are usually combined into differentiating models. Furthermore, since most individual VOCs are aspecific it is preferred to use VOC panels or differentiating models rather than individual compounds to classify patients (10).

Since studies in breath of IBS patients are still sparse, the aim of this study is to analyze and compare VOC profiles in both breath and fecal samples of IBS patients and HCs using the MCC/IMS methodology.

## Methodology

### Study population

Patients with IBS and HCs were recruited via the tertiary referral motility clinic of the department of Gastroenterology and Hepatology of the Antwerp University Hospital (UZA), via the University of Antwerp, and a patient-centered informative website.^1^ IBS patients were only included if they fulfilled the Rome IV criteria for IBS. Exclusion criteria for both patients and HC were the presence of IBD, celiac disease, any history of malignancy in the gastrointestinal tract, pregnancy, and breastfeeding.

HC were also excluded if they experienced any gastrointestinal complaints. Patients were further categorized according to their dominant stool pattern: diarrhea (IBS-D), constipation (IBS-C), or mixed (IBS-M) (22). No patients with the unspecified subtype (IBS-U) were included. A general profile with biometrics, medical history, and medication list was compiled. Additional information was collected through digital questionnaires at time of inclusion [IBS-symptom severity system (IBS-SSS), Hospital Anxiety and Depression Scale (HADS), visceral sensitivity index (VSI), irritable bowel syndrome quality of life questionnaire (IBS-QOL), food diary, and exercise habits]. After inclusion, breath and fecal samples were collected within the same week. Samples were collected between August 2019 and April 2021.

All participants gave written informed consent approved by the Ethics Committee of the Antwerp University Hospital (19/38/419). Samples were registered and stored until analysis in the “Biobank Antwerpen,” Antwerp, Belgium (ID: BE 71030031000). The study was performed according to the Helsinki declaration (23).

### Sampling of breath

A BioScout breath analyzing device (B&S Analytik, Dortmund, Germany) operating on VOCan v2.7 software was used for breath sampling. Details regarding the set-up are shown in Table 1. This device consists of a Breath Discovery ion mobility spectrometer coupled to a MCC, which is connected to a SpiroScout ultrasound-controlled breath sampler (Ganshorn Medizin Electronic, Niederlauer, Germany) by a sample loop. Participants were asked to refrain from eating, drinking, brushing their teeth, taking medication, and smoking at least 2 h before breath sampling. Patients were then asked to rinse their mouth with distilled water, put on a nose-clip and breathe tidally for 3 min through the SpiroScout sampler connected to a bacteria filter. Subsequently, 10 mL of alveolar air was collected and immediately analyzed in positive mode. After breath sampling, a patient-related background sample was collected to correct for potential environmental contamination.

**TABLE 1:** Multicapillary column/ion mobility spectrometry (MCC-IMS) characteristics.

Parameter	
Ionization source	^63^Ni (95 MBq)
Electrical field strength	320 V/cm
Length of the drift region	12 cm
Diameter of the drift region	15 mm
Length of the ionization chamber	15 mm
Shutter opening time	300 μs
Shutter impulse time	100 ms
Drift gas	α_2_-nitrogen gas
Drift gas flow	100 ml/min
Carrier gas flow	100 ml/min
Working temperature	Ambient temperature
Pressure	Ambient pressure (101 kPa)
Pre-separation	Multi-capillary column OV-5, polar, 1,000 packed columns, 3 mm diameter (Multichrom Ltd., Novosibirsk, Russia)
Column temperature	40°C, isothermal, adjusted
Tubing	PTFE

### Sampling of feces

Fecal samples were collected in a plastic container in the same week as the breath samples, preferably on the same day. Participants were asked to hand in the fecal sample within 4 h after defecation after which the sample was aliquoted and stored at −80°C without the addition of any buffers. Samples were left to defrost overnight at 4°C before analysis. For the fecal analysis, a BioScout breath analyzing device operating on VOCan v4.1 software was used (B&S Analytik, Dortmund, Germany). The sample loop of the MCC/IMS was connected to a custom-made stainless-steel IMS-box (24). In this closed box, 0.5 grams of feces was heated at 37°C for 1 h. Subsequently, the IMS box was flushed with nitrogen gas (α_1_-nitrogen gas; 99.999% pure; Air Liquide Medical, Schelle, Belgium), sampling 10 mL of headspace air followed by immediate analysis by MCC/IMS in positive mode. Background samples of the empty set-up were collected to correct for potential environmental and instrumental contamination.

### Data handling

**FIGURE 1:**
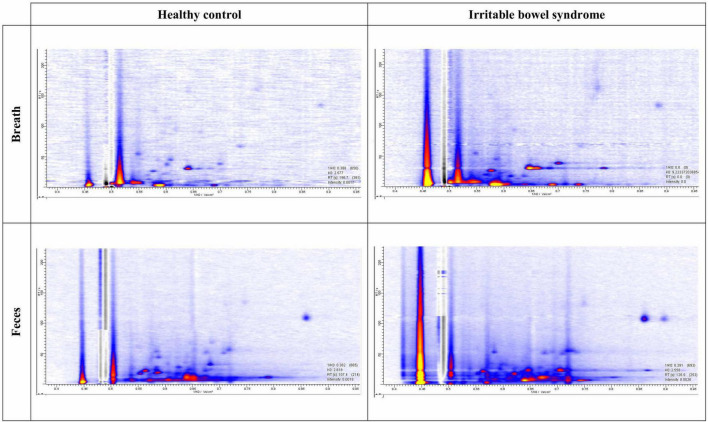
Example of chromatograms. Intensities are presented with the help of a color code from low intensity (blue) to high intensity (yellow).

All MCC/IMS data were analyzed using VisualNow Software v3.9 (B&S Analytik, Dortmund, Germany) as previously described (25, 26). In short, the raw IMS chromatograms were denoised through baseline correction using a low pass filter and aligned. Next, data were normalized to the reactant ion peak (RIP) and RIP-tailing was compensated by subtracting a median spectrum from each chromatogram within the data set. Further, the data was smoothed, and the chromatograms were visually inspected for the presence of VOCs. An example of a chromatogram can be found in Figure 1. If a VOC was present in either breath/fecal or background sample, they were manually selected and analyzed, resulting in a list of VOC peak intensities (maximum peak height in the selected peak area). For every VOC, the alveolar gradient was calculated by subtracting the intensity of the VOC in the background sample from the intensity of the VOC in the corresponding breath/fecal sample. These gradients were used as independent variables for further statistical analysis. Fecal VOC gradients are shown as “PF” and breath VOC gradients as “PB.”

### Statistical analysis

Categorical patient characteristics are expressed as *n* (%) and compared using Pearson’s χ^2^ test, followed by pairwise comparisons between the subpopulations with a Bonferroni correction for multiple comparisons. Continuous variables are expressed as median (range) and comparisons between groups were made using the Kruskal–Wallis test, followed by Dunn’s *post-hoc* test with a Bonferroni adjustment for multiple comparisons. All analyses were performed using R (version 4.1.1) in RStudio (version 1.4.1717). A significance level of α = 0.05 was used throughout the analysis.

Logistic least absolute shrinkage and selection operator (lasso) regression with leave-one-out cross-validation (LOOCV) was used to select the VOCs that best differentiated IBS patients from HC, or IBS subtypes from other subtypes. Besides VOCs, the clinical characteristics age and gender were included as potential predictors to prevent confounding. Models were fitted using the R package *glmnet* (version 4.1-2). The alveolar gradient of these VOCs selected by the lasso regression in breath, feces, or in models containing VOCs from both matrices were then used as independent variables in a logistic regression model which was internally validated by LOOCV. To prevent overfitting of the data, a maximum of two predictors was considered in each logistic model. Using the predicted outcome of all subjects, receiver operating characteristic (ROC) curves were generated. Relevant cut-off values were chosen to optimize accuracy and the model’s sensitivity and specificity were estimated based upon these cut-off values. The accuracy of classification models based on VOCs derived from feces, breath, or a combination of both was compared by testing for a difference in the AUC of the respective ROC curves using a bootstrapping approach, as implemented by the pROC package (version 1.18.0). The AUC is a different performance characteristic compared to accuracy and does not depend on the earlier mentioned cut-off values but can be considered as a general indicator of the discriminatory capacity of the model.

To explore the impact of differences in the IBS phenotype on the VOC profiles, the IBS population was stratified based on the presence of depression (HADS score > 8 on depression subscale), anxiety (HADS score > 8 on anxiety subscale), use of antibiotics, use of probiotics, symptom severity, quality of life, and visceral sensitivity. For continuous variables, the stratification was based on the median value in the population. Logistic lasso regression models were then used to generate VOC profiles that differentiated the two IBS subpopulations.

## Results

### Study population

**FIGURE 2:**
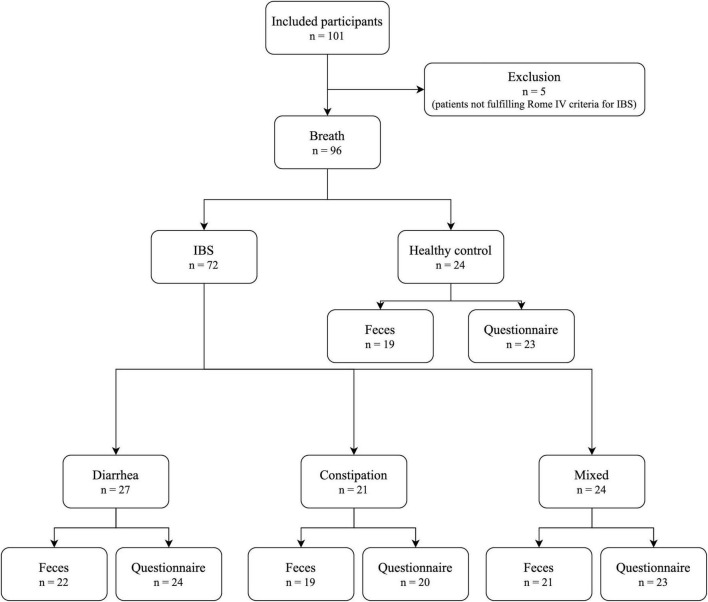
Flow of study participants; IBS, irritable bowel syndrome.

In total, 101 subjects were included (Figure 2). Five patients did not meet Rome IV criteria and were excluded, leaving 96 participants for breath sampling (24 HC; 27 IBS-D; 21 IBS-C; and 24 IBS-M). Of these, 81 participants had a matched fecal sample (19 HC; 22 IBS-D; 19 IBS-C; and 21 IBS-M). Baseline characteristics of the study population are shown in Table 2. There were no statistically significant differences between the groups for age, gender, or BMI. There was, however, a trend for a more female predominant population in the IBS groups compared to controls. IBS patients had significantly higher scores on IBS-SSS, VSI, and IBS-QOL compared to controls, with no differences between the subtypes. In addition, IBS-M patients had a significantly higher HADS score for anxiety compared to controls, while IBS-D and IBS-C were not significantly different from HC.

**TABLE 2:** Baseline characteristics.

	HC	IBS-D	IBS-C	IBS-M	*p*-value
**Number**	24	27	21	24	
**Gender Males:Females**	11:13	3:24	5:16	4:20	*p* = 0.0567^§^
**Age (years; range)*^a^***	27 (18–70)	37 (18–78)	38 (20–77)	32 (23–64)	*p* = 0.2161^§^
**BMI (kg/m^2^; range)*^a^***	22.7 (18.4–29.7)	22.9 (18.7–37.5)	22.6 (16.8–33.5)	25.0 (19.5–39.1)	*p* = 0.5032^§^
**IBS-SSS (range)*^a^***	4 (0–92)	220 (68–400)	251 (135–398)	264 (61–383)	*p* < 0.0001^§ &^
**VSI (range)*^a^***	9 (0–40)	24.5 (5–62)	27 (0–70)	28 (1–55)	*p* = 0.0005^§ &^
**Median IBS-QOL (range)*^a^***	20 (19–33)	48 (22–78)	50 (24–78)	52 (22–97)	*p* < 0.0001^§ &^
**Positive HADS-An (%)**	4/23 (17%)	13/24 (54%)	10/19 (53%)	16/23 (70%)	*p* = 0.0038^¶ $^
**Positive HADS-Dep (%)**	1/23 (4%)	2/24 (8%)	4/19 (21%)	6/23 (26%)	*p* = 0.1238^¶^
**Antibiotic use (%)**	1/24 (4%)	5/27 (19%)	6/21 (29%)	2/24 (8%)	*p* = 0.0906^¶^
**Probiotic use (%)**	1/24 (4%)	8/27 (30%)	6/21 (29%)	6/24 (25%)	*p* = 0.1082^¶^

An, anxiety; C, constipation; D, diarrhea; Dep, depression; GI, gastrointestinal; HADS, hospital anxiety and depression score; HC, healthy control; IBS, irritable bowel syndrome; IBS-SSS, symptom severity score; IBS-QOL, quality of life; M, mixed; SD, standard deviation; VSI, visceral sensitivity index.

*^a^*Median (range).

^§^Median (range) with Kruskal–Wallis test, followed by Dunn’s *post-hoc* test with a Bonferroni adjustment for multiple comparisons.

^¶^n (%) with Pearson’s χ^2^ test, followed by pairwise comparisons between the subpopulations with a Bonferroni correction for multiple comparisons.

^&^Significant differences between HC and each of the patient subtypes, not amongst patient subtypes.

^$^Significant difference between HC and IBS-M.

### Volatile analysis

**FIGURE 3:**
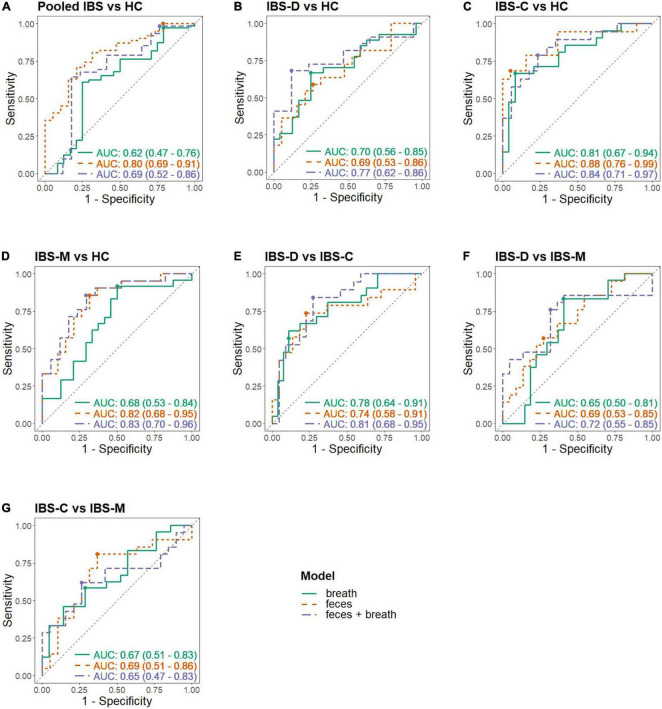
Receiver operating characteristic (ROC) curves in different matrices. Each panel shows the ROC curve of the breath, fecal, and combined model; **(A)** Pooled IBS patients vs. HC, **(B)** IBS-D vs. HC, **(C)** IBS-C vs. HC, **(D)** IBS-M vs. HC, **(E)** IBS-D vs. IBS-C, **(F)** IBS-D vs. IBS-M, **(G)** IBS-C vs. IBS-M; -C, constipation; -D, diarrhea; HC, healthy control; IBS, irritable bowel syndrome; -M, mixed.

In total, 92 and 211 VOCs were identified in respectively breath (PB) and fecal (PF) samples (Supplementary Tables 1, 2). Supplementary Table 3 summarizes the VOCs selected by the lasso regression analysis followed by LOOCV. Figure 3 demonstrates the ROC curves in the different matrices. We first determined whether it was feasible to differentiate IBS patients from HCs based upon VOCs in breath and fecal samples using MCC/IMS.

#### Pooled irritable bowel syndrome patients vs. healthy controls

The fit and internal validation of the logistic regression models and the characteristics of the VOCs used in these models are shown in Tables 3, 4, respectively. In breath, IBS patients were differentiated from HC with an AUC of 0.62 (0.47–0.76), 97.2% (91.2–99.5%) sensitivity, and 20.8% (8.1–40.3%) specificity. Based upon fecal VOCs, a higher AUC was obtained [0.80 (0.69–0.91)] with a specificity of 21.1% (7.1–43.3%), and a sensitivity of 100% (95.3–100%). Lastly, combining the breath and fecal VOC matrices into one model averaged the performance of the individual matrices, resulting in an AUC of 0.69 (Table 3; Figure 3).

**TABLE 3:** Differentiating models based on VOC pattern.

	Pooled IBS vs. HC	IBS-D vs. HC	IBS-C vs. HC	IBS-M vs. HC	IBS-D vs. IBS-C	IBS-C vs. IBS-M	IBS-D vs. IBS-M
**Breath**							
Sens% (95% CI)	97.2 (91.2–99.5)	66.7 (47.6–82.4)	66.7 (44.9–84.1)	91.7 (75.1–98.6)	57.1 (35.8–76.7)	58.3 (38.3–76.5)	83.3 (64.6–94.5)
Spec% (95% CI)	20.8 (8.1–40.3)	75.0 (55.1–89.2)	91.7 (75.1–98.6)	50.0 (30.6–69.4)	88.9 (72.7–97.1)	71.4 (49.8–87.5)	59.3 (40.3–76.4)
Acc% (95% CI)	78.1 (69.1–85.5)	70.6 (57.1–81.8)	80.0 (66.5–89.8)	70.8 (56.9–82.3)	75.0 (61.4–85.7)	64.4 (49.8–77.3)	70.6 (57.1–81.8)
AUC (95% CI)	0.62 (0.47–0.76)	0.70 (0.56–0.85)	0.81 (0.67–0.94)	0.68 (0.53–0.84)	0.78 (0.64–0.91)	0.67 (0.51–0.83)	0.65 (0.50–0.81)
**Feces**							
Sens% (95% CI)	100 (95.3–100)	59.1 (38.1–77.9)	68.4 (45.5–86.1)	85.7 (65.9–96.2)	73.7 (51.0–89.6)	81.0 (60.2–93.6)	57.1 (35.8–76.7)
Spec% (95% CI)	21.1 (7.1–43.3)	73.7 (51.0–89.7)	94.7 (76.7–99.7)	68.4 (45.5–86.1)	77.3 (56.6–91.2)	63.2 (40.4–82.2)	72.7 (51.7–88.1)
Acc% (95% CI)	81.5 (72.0–88.8)	65.9 (50.5–79.1)	81.6 (67.0–91.6)	77.5 (62.7–88.4)	75.6 (60.9–86.9)	72.5 (57.3–84.6)	65.1 (50.1–78.2)
AUC (95% CI)	0.80 (0.69–0.91)	0.69 (0.53–0.86)	0.88 (0.76–0.99)	0.82 (0.68–0.95)	0.74 (0.58–0.91)	0.69 (0.51–0.86)	0.69 (0.53–0.85)
**Breath and feces**							
Sens% (95% CI)	98.4 (92.3–99.9)	68.2 (47.0–84.9)	78.9 (56.7–92.9)	85.7 (65.9–96.2)	84.2 (62.8–95.8)	61.9 (40.3–80.5)	76.2 (54.9–90.7)
Spec% (95% CI)	23.5 (8.0–47.5)	88.2 (66.3–98.0)	76.5 (52.5–92.0)	70.6 (46.4–88.3)	72.7 (51.7–88.1)	73.7 (51.0–89.6)	68.2 (47.0–84.9)
Acc% (95% CI)	82.3 (72.7–89.5)	76.9 (61.9–88.1)	77.8 (62.2–89.1)	78.9 (63.9–89.7)	78.0 (63.6–88.7)	67.5 (52.0–80.6)	72.1 (57.4–83.9)
AUC (95% CI)	0.69 (0.52–0.86)	0.77 (0.62–0.92)	0.84 (0.71–0.97)	0.83 (0.70–0.96)	0.81 (0.68–0.95)	0.65 (0.47–0.83)	0.72 (0.55–0.88)

Acc, accuracy; AUC, area under the curve; C, constipation; CI, confidence interval; D, diarrhea; HC, healthy control; IBS, irritable bowel syndrome; M, mixed; Sens, sensitivity; Spec, specificity; VOC, volatile organic compound.

**TABLE 4:** Characteristics VOCs used in logistic regression models.

VOC	Model		1/K0	RT	1/K0 radius	RT radius
**Breath**						
PB0	IBS-C vs. HC*	IBS-C vs. IBS-M*	0.770	161.5	0.006	5.5
PB1	Quality of life	Quality of life*	0.739	67.4	0.006	2.3
PB2	IBS-D vs. IBS-M	Probiotics	0.757	30.2	0.005	1.7
PB11	IBS-D vs. HC IBS-D vs. IBS-M	IBS-D vs. HC*	0.684	30.2	0.005	2.1
PB12	IBS-D vs. IBS-C*		0.704	30.0	0.007	1.7
PB14	Quality of life	Quality of life*	0.619	93.1	0.006	3.2
PB23	Visceral sensitivity		0.502	6.9	0.006	2.0
PB28	Anxiety	Anxiety*	0.609	8.5	0.006	1.8
PB29	Antibiotics*		0.530	11.5	0.005	1.6
PB45	IBS-C vs. HC IBS-C vs. IBS-M	IBS-C vs. IBS-M*	0.666	13.6	0.005	2.1
PB57	IBS-M vs. HC		0.707	43.7	0.005	1.6
PB61	IBS-C vs. HC IBS-D vs. IBS-C	IBS-C vs. IBS-M IBS-C vs. HC*	0.558	18.6	0.003	1.9
PB63	Antibiotics		0.550	7.5	0.004	1.5
PB66	Visceral sensitivity		0.603	37.8	0.004	1.6
PB74	Depression		0.597	64.8	0.009	2.3
PB77	Anxiety Anxiety*	Visceral sensitivity*	0.884	37.3	0.008	1.0
PB78	Depression Symptom severity	Depression*	0.640	157.7	0.007	4.8
PB81	Pooled IBS vs. HC Pooled IBS vs. HC*	Symptom severity	0.810	3.0	0.004	2.0
PB89	IBS-D vs. IBS-C	IBS-D vs. IBS-C*	0.625	30.6	0.005	1.4
**Feces**						
PF0	Depression		0.859	108.2	0.005	4.6
PF3	Antibiotics		0.548	5.0	0.007	1.1
PF9	IBS pooled vs. HC IBS-M vs. HC	IBS-M vs. HC*	0.528	0.5	0.006	0.7
PF10	Anxiety Visceral sensitivity	Visceral sensitivity*	0.571	4.1	0.004	1.6
PF32	IBS-D vs. IBS-M		0.588	19.2	0.004	1.5
PF37	IBS-D vs. IBS-M		0.451	9.1	0.003	2.6
PF43	IBS-D vs. IBS-C		0.662	9.1	0.005	1.1
PF56	IBS pooled vs. HC IBS-D vs. HC IBS-C vs. HC	IBS-M vs. HC IBS-M vs. HC*	0.684	31.6	0.008	2.0
PF63	IBS-D vs. IBS-M*		0.583	14.1	0.005	1.6
PF64	IBS-D vs. IBS-M*		0.650	2.5	0.007	1.5
PF65	Quality of life		0.670	28.2	0.003	3.8
PF74	Probiotics		0.528	9.1	0.005	0.8
PF80	Anxiety		0.554	17.5	0.004	1.2
PF84	Quality of life		0.572	9.0	0.006	1.1
PF87	Symptom severity	Symptom severity*	0.573	0.0	0.004	1.0
PF92	IBS-D vs. HC		0.640	13.5	0.003	1.3
PF116	Depression	Depression*	0.539	11.2	0.003	2.7
PF170	IBS-C vs. HC		0.573	64.0	0.004	1.8
PF172	IBS-D vs. IBS-C		0.583	24.8	0.004	1.7
PF207	Symptom severity	Symptom severity*	0.708	21.5	0.003	0.9
PF208	IBS-C vs. IBS-M	Probiotics*	0.578	18.5	0.003	3.1

C, constipation; CI, confidence interval; D, diarrhea; HC, healthy control; IBS, irritable bowel syndrome; M, mixed; PB, breath volatile; PF, fecal volatile; VOC, volatile organic compound.

*Combination model.

The differences in performance (in terms of AUC) were not significantly different. However, there was a trend when comparing breath and fecal models with a higher performance in the feces-based classifier (breath vs. feces: *p* = 0.054; breath vs. combination: *p* = 0.541; feces vs. combination: *p* = 0.284).

#### Irritable bowel syndrome subtypes vs. healthy controls

Most of the models differentiating the individual IBS subtypes with HC appear less optimal than the pooled IBS patient models, as shown in Table 3. In breath, the best classification was found when differentiating IBS-C patients from HC with a specificity of 91.7% (75.1–98.6%), sensitivity of 66.7% (44.9–84.1%), and AUC of 0.81 (0.67–0.94). In feces, this differentiation between IBS-C and HC was possible with 94.7% (76.7–99.7%) specificity, 68.4% (45.5–86.1%) sensitivity, and an AUC of 0.88 (0.76–0.99). This fecal model was based upon the VOCs PF56 and PF170, the former one was also included in the pooled IBS model. Pooling VOCs of both matrices resulted in a similar outcome (Table 3; Figure 3).

Irritable bowel syndrome diarrhea patients were better differentiated by breath VOCs 0.70 (0.56–0.85) AUC, 66.7% (47.6–82.4%) sensitivity, and 75.0% (55.1–89.2%) specificity. In feces, differentiation resulted in an AUC of 0.69 (0.53–0.86) and in the model combining both VOCs the resulting AUC was the highest at 0.77 (0.62–0.92).

Differentiating IBS-M patients and HC in breath resulted in an AUC of 0.68 (0.53–0.84). Again, fecal VOCs performed better, based upon the VOCs PF9 and PF56 from the pooled IBS model with an AUC of 0.82 (0.68–0.95), similar as the combined model [AUC 0.83 (0.70–0.96)].

Given that IBS is a heterogenous disorder with a multifactorial etiology, we hypothesized that VOC profiles might also differ between the IBS subtypes mutually. Therefore, the role of volatomics in differentiating IBS subtypes from each other was investigated (Table 3; Figure 3).

#### Differentiating irritable bowel syndrome patient subtypes

In breath, the best classification was obtained by comparing IBS-D and IBS-C patients, resulting in an AUC of 0.78 (0.64–0.91) [specificity of 88.9% (72.7–97.1%), sensitivity of 57.1% (35.8–76.7%)]. This was amongst other things based upon PB61 which was also found to differentiate between IBS-C and IBS-M patients, and IBS-C vs. HC. Differentiating IBS-M patients from IBS-D and IBS-C patients resulted in lower AUCs [respectively 0.65 (0.50–0.81) and 0.67 (0.51–0.83)]. When focusing on fecal volatiles AUCs ranged between 0.69 and 0.74 (Table 3; Figure 3). There was no overlap in fecal VOCs when subtyping IBS-patients or when different IBS subtypes were compared to HC.

When volatiles from both breath and feces were combined, the classifier’s performance for IBS-C vs. IBS-M was 0.65 (0.47–0.83), specificity of 73.7% (51.0–89.6%), and sensitivity of 61.9% (40.3–80.5%). An acceptable differentiation was found when comparing IBS-D with IBS-C patients, characterized by an AUC of 0.81 (0.68–0.95), 72.7% (51.7–88.1%) specificity, and 84.2% (62.8–95.8%) sensitivity, and when comparing IBS-D to IBS-M patients, resulting in an AUC of 0.72 (0.55–0.88), 76.2% (54.9–90.7%) sensitivity, and 68.2% (47.0–84.9%) specificity.

In general, an acceptable differentiation of IBS patients from HC was observed, as well as between subtypes based upon different VOCs. Subsequently, we wanted to evaluate the influence of other clinical characteristics on VOC profiles to further elucidate the between-patient variability. Therefore, patients were reclassified based on clinical characteristics (Table 5). For these analyses HC were not included.

**TABLE 5:** Differentiating models based on clinical characteristics in patients.

	Psychological comorbidities		Microbiota influencing therapies		Symptom scores		
	Depression	Anxiety	Antibiotics	Probiotics	Quality of life	Visceral sensitivity	Symptom severity
**Breath**							
Sens% (95% CI)	44.4 (16.1–75.9)	84.8 (69.6–94.2)	53.8 (27.5–78.7)	52.9 (29.8–75.2)	86.2 (70.0–95.4)	62.1 (43.7–78.2)	89.4 (78.0–96.0)
Spec% (95% CI)	93.9 (84.3–98.4)	40.0 (22.4–59.8)	95.9 (87.2–99.3)	86.7 (74.4–94.4)	55.2 (37.1–72.3)	72.4 (54.3–86.3)	36.4 (12.8–66.3)
Acc% (95% CI)	86.2 (75.5–93.4)	65.5 (52.7–76.9)	87.1 (77.0–93.8)	77.4 (65.8–86.5)	70.7 (58.1–81.3)	67.2 (54.5–78.4)	79.3 (67.5–88.3)
AUC (95% CI)	0.64 (0.39–0.90)	0.66 (0.52–0.80)	0.79 (0.63–0.95)	0.70 (0.54–0.86)	0.69 (0.55–0.83)	0.66 (0.51–0.80)	0.56 (0.32–0.81)
**Feces**							
Sens% (95% CI)	33.3 (9.3–66.7)	75.8 (59.1–88.0)	23.1 (6.3–50.8)	47.1 (24.8–70.3)	69.0 (50.7–83.7)	58.6 (40.3 - 75.3)	95.7 (86.7–99.3)
Spec% (95% CI)	98.0 (90.4–99.9)	56.0 (36.5–74.2)	95.9 (87.2–99.3)	88.9 (77.1–95.8)	58.6 (40.3–75.3)	62.1 (43.7–78.2)	18.2 (3.2–48.3)
Acc% (95% CI)	87.9 (77.6–94.6)	67.2 (54.5–78.4)	80.6 (69.5–89.1)	77.4 (65.8–86.5)	63.8 (50.9–75.3)	60.3 (47.4–72.3)	81.0 (69.5–89.6)
AUC (95% CI)	0.78 (0.61–0.95)	0.66 (0.52–0.81)	0.75 (0.58–0.91)	0.72 (0.57–0.87)	0.61 (0.46–0.76)	0.57 (0.42–0.72)	0.66 (0.48–0.85)
**Breath and feces**							
Sens% (95% CI)	55.6 (24.1–83.9)	60.6 (43.4–76.0)	46.2 (21.3–72.6)	52.9 (29.8–75.2)	86.2 (70.0–95.4)	69.0 (50.7–83.7)	95.7 (86.7–99.3)
Spec% (95% CI)	95.9 (87.2–99.3)	68.0 (48.2–83.9)	83.7 (71.4–92.1)	95.6 (86.1–99.2)	55.2 (37.1–72.3)	62.1 (43.7–78.2)	18.2 (3.2–48.3)
Acc% (95% CI)	89.7 (79.8–95.7)	63.8 (50.9–75.3)	75.8 (64.1–85.2)	83.9 (73.2–91.5)	70.7 (58.1–81.3)	65.5 (52.7–76.9)	81.0 (69.5–89.6)
AUC (95% CI)	0.76 (0.54–0.98)	0.66 (0.52–0.80)	0.73 (0.57–0.89)	0.75 (0.59–0.91)	0.69 (0.55–0.83)	0.64 (0.49–0.78)	0.66 (0.48–0.85)

Acc, accuracy; AUC, area under the curve; CI, confidence interval; Sens, sensitivity; Spec, specificity.

#### Analysis based on clinical characteristics other than stool pattern

##### Psychological comorbidities

Thirty-nine out of the 66 IBS patients that completed the HADS questionnaire (59.1%) scored positive for anxiety and were compared with patients scoring negative for anxiety. Both breath and fecal analysis allowed a modest differentiation between both groups with an AUC of 0.66 (Table 5). None of the contributing VOCs used in the logistic regression were found to overlap with the differentiation between subtypes (Table 5). Combining the VOCs of both matrices did not improve the differentiating capacity [AUC 0.66 (0.52–0.80)].

Twelve out of 66 IBS patients (18.2%) scored positive for depression, and all patients scoring positive for depression also scored positive for anxiety, making this a subpopulation of the previous analysis. Based upon breath volatiles, a differentiation between depressed and non-depressed patients was found with an AUC of 0.64 (0.39–0.90) [specificity of 93.9% (84.3–98.4%), sensitivity of 44.4% (16.1–75.9%)]. In feces, the AUC was higher at 0.78 (0.61–0.95) [33.3% (9.3–66.7%) sensitivity, 98.0% (90.4–99.9%) specificity] and, as with anxiety, none of the contributing VOCs were found to overlap with the differentiation between subtypes. The combined model performed similar with a specificity of 95.9% (87.2–99.3%), sensitivity of 55.6% (24.1–83.9%), and an AUC of 0.76 (0.54–0.98).

##### Microbiota influencing therapies

Thirty IBS patients (41.7%) used antibiotics and/or probiotics in the 3 months prior to sample collection. Patients using antibiotics (13/72, 18.1%) were differentiated by breath volatiles from those not using antibiotics with an AUC of 0.79 (0.63–0.95), 95.9% (87.2–99.3%) specificity, and 53.8% (27.5–78.7%) sensitivity. Based upon fecal volatiles, the AUC was 0.75 (0.58–0.91) [23.1% sensitivity (6.3–50.8%) and 95.9% (87.2–99.3%) specificity]. In the combined model, the classifier’s performance was lower [AUC 0.73 (0.57–0.89)].

Patients on probiotics (20/72, 27.8%) were accurately differentiated from those not using probiotics based upon VOCs in breath [AUC 0.70 (0.54–0.86)], feces [AUC 0.72 (0.57–0.87)], or both combined [AUC 0.75 (0.59–0.91)].

##### Symptom scores

Irritable bowel syndrome patients were also differentiated based on the scores of the IBS-QOL questionnaire, IBS-SSS, and VSI (Table 5). Based upon volatiles in breath, patients with a high score on IBS-QOL could be differentiated from those with a low score with an AUC of 0.69 (0.55–0.83). Fecal volatiles on the other hand, were not as performant [AUC of 0.61 (0.46–0.76)]. Combining both breath and fecal VOCs, groups resulted in the same differentiating values as breath models [AUC 0.69 (0.55–0.83)]. Also, no VOCs were found to be overlapping with those subtyping IBS patients.

Furthermore, patients with a high VSI could be differentiated from patients with a low VSI in breath, feces, and combined models [respectively AUCs of 0.66 (0.51–0.80), 0.57 (0.42–0.72), 0.64 (0.49–0.78)].

Differentiating patients based on symptom severity (IBS-SSS) performed similar with AUCs ranging between 0.56 and 0.66, albeit at the cost of low specificities.

## Discussion and conclusion

Research and clinical practice are still eagerly awaiting the discovery of biomarkers to diagnose and characterize patients with IBS. A recent development in this area is the field of volatomics studying VOCs (10).

A recent systematic review from our group stressed that current volatomics research in IBS is heterogenous and limited to mostly fecal analysis, however, results stressed promising clinical applications (10). Our study is the first to demonstrate the use of VOCs in diagnosing and subtyping patients with IBS in breath and fecal samples using the more clinically applicable MCC/IMS. The results of our models are in line with previous studies using GC-MS (11–18). When differentiating IBS patients from HC, fecal volatiles performed better compared to breath. However, as there are limited differences in differentiating values, breath sampling could be preferred given the ease of providing and analyzing a sample. This is further strengthened by the fact that both breath and fecal volatiles comparably differentiated each IBS subtype from HC. However, combining volatiles of both biological samples showed no added value in this feasibility study. The highest classification characteristics across all matrices were observed when pooling IBS subtypes and comparing them to HC (AUC of 0.80 vs. 0.62). This showed the feasibility of the method and the presence of different VOCs between HC and symptomatic patients with IBS and suggests that VOC measurement and identification could possibly evolve into a clinically useful biomarker. However, when comparing individual IBS subtypes (IBS-D, IBS-C, and IBS-M) with HC, the differentiating potential was found to be slightly lower. This is somewhat unexpected as we predicted that subtyping patients based on their dominant stool pattern (confer Rome IV criteria) would increase the ability of VOCs to differentiate patients from HC, because of underlying differences in pathophysiology. Still, when we differentiated these patient subtypes from each other, results were also acceptable. One VOC in breath, PB61, was found as an important classifier when differentiating IBS-C patients from IBS-D, IBS-M, and HC, possibly linking its presence to the IBS-C subtype. There was little to no overlap in VOCs when subtyping IBS patients or when subtypes were compared to HC, which could suggest the existence of subtype-specific volatiles that could relate to the dominant stool pattern. However, IBS patients could also be differentiated based on clinical characteristics other than stool pattern. VOCs that contributed to these differentiations were different from those allowing subtyping of IBS patients, suggesting that other parameters could play a role in the differentiation or subtyping of IBS patients.

Since the microbiota are omnipresent in the human colon and feces, and produces specific VOCs (27), it is expected that the microbial composition would be better reflected in fecal VOC profiles compared to breath profiles. However, while the classification models differentiating IBS patients using probiotics vs. IBS patients not using probiotics had a similar performance in feces and breath (AUC of 0.72 and 0.70), the models using antibiotics had a slightly better performance when using breath (AUC of 0.75 vs. 0.79). Hence, the microbiota has an influence on VOC composition in general and these VOCs could reflect a change in microbial composition. This has also been demonstrated by Smolinska et al. (27) in patients with Crohn’s disease and Sagar et al. (19) in patients with bile acid diarrhea and IBS-D. However, more research is needed to further elucidate the origin and relationship between the microbiota on the one hand and its manipulation using medication and VOC profiles on the other hand.

Psychological characteristics like the presence of depression or anxiety had a higher differentiating ability in feces compared to breath models. This could be explained by the differences in underlying pathophysiology and metabolism in IBS patients with comorbid anxiety or depression and the role of the gut-brain-microbiome axis (28). This is in accordance with the recent publications of Black et al. (29, 30) demonstrating that classifying patients based on psychological burden had a higher stability over time compared to a classification based on dominant stool pattern.

Breath and fecal volatiles were also able to differentiate patients based on questionnaires assessing symptom severity, quality of life, and visceral sensitivity. Considering that those clinical characteristics, other than dominant stool pattern, are able to accurately differentiate patients demonstrates the importance of questioning these characteristics and maybe consider alternative classifications of IBS patients. The heterogeneity of IBS, both in clinical presentation and underlying pathophysiology, further demonstrates the need to thoroughly characterize patients when looking for novel biomarkers.

Despite these positive findings, our study did have limitations which should be considered for future research. First, this feasibility study had a moderate sample size that needs to be increased in future validation trials. As a result, despite the use of lasso regression in order to avoid overfitting of the data, the analysis based on clinical characteristics oftentimes involved a small sample size which could still lead to overfitting and overoptimistic results. Secondly, only HC and IBS patients were included as the intent was to explore the feasibility of sampling and analyzing VOCs by MCC/IMS in these populations. When further investigating the role of volatomics in clinical practice, large-scale studies should be initiated enclosing other common gastrointestinal disorders, such as celiac disease and IBD, since these are important differential diagnoses of IBS. If VOCs can differentiate IBS from these organic disorders, they could be further developed into a diagnostic biomarker test, which is one of the major unmet needs in IBS management.

Thirdly, we only collected samples at a single time point. Little is known about the natural evolution of VOC profiles over time. Hence, long-term follow-up of a patient population to evaluate spontaneous fluctuation and the impact of specific therapies on VOC profiles will help understand and optimize the current classification models.

We also did not record the consistency of the fecal samples and it is currently unknown if stool consistency *per se* has an influence on VOC output during measurement. Nevertheless, further optimization of fecal VOC analysis, taking stool consistency into account, is advised.

In conclusion, we demonstrated the potential of VOCs in the characterization of patients with IBS. VOCs accurately differentiated IBS patients from HC. In addition, independent VOCs were found to differentiate IBS patients when classified into the classical subtypes based on their dominant stool pattern (Rome IV criteria) compared to controls. Furthermore, volatiles were able to distinguish patients based on clinical characteristics, other than their dominant stool pattern, such as psychological states, symptom scores, and microbiota-influencing treatment, suggesting the possibility of alternative subtyping of IBS patients. We therefore plead for the inclusion of other clinical characteristics when developing biomarkers for IBS in general and using volatomics in particular. The results of this study should be validated in a larger population including an extensive clinical characterization of patients and microbiota analysis.

## Data availability statement

The original contributions presented in this study are included in the article/Supplementary material, further inquiries can be directed to the corresponding author.

## Ethics statement

The studies involving human participants were reviewed and approved by Ethics Committee of the Antwerp University Hospital. The patients/participants provided their written informed consent to participate in this study.

## Author contributions

KVM, BDW, HC, JDM, HDS, and KL concepted the study. KVM, HDS, HL, TH, and SVM recruited the patients and collected the samples. KVM, HL, TH, and SVM performed the breath analysis. KVM performed the fecal analysis and drafted the manuscript. KVM, KL, and NH analyzed the data and performed the statistical analysis. All authors have read and approved the final version.

## Footnotes

1
www.ibsbelgium.org/
