# Supplementary material for: Volatile organic compound profiling as a potential biomarker in irritable bowel syndrome: A feasibility study
Source: Front Med (Lausanne). 2022 Aug 4;9:960000. doi: 10.3389/fmed.2022.960000 (PMC9388331; doi:10.3389/fmed.2022.960000)
Supplement: Supplementary file 1 [file Table_1.DOCX]

Supplementary materials

[Supplementary table 1: Detected volatile organic compounds in breath samples 2](#_Toc103255027)

[Supplementary table 2: Detected volatile organic compounds in fecal samples 3](#_Toc103255028)

[Supplementary table 3: Selected volatile organic compounds by lasso regression 6](#_Toc103255029)

# **Supplementary table 1: Detected volatile organic compounds in breath samples**

| **VOC** | **1/K0** | **RT** | **1/K0 radius** | **RT radius** |  | **VOC** | **1/K0** | **RT** | **1/K0 radius** | **RT radius** |
| --- | --- | --- | --- | --- | --- | --- | --- | --- | --- | --- |
| **PB0** | 0·770 | 161·5 | 0·006 | 5·5 |  | **PB46** | 0·643 | 34·5 | 0·005 | 1·3 |
| **PB1** | 0·739 | 67·4 | 0·006 | 2·3 |  | **PB47** | 0·886 | 134·5 | 0·008 | 3·4 |
| **PB2** | 0·757 | 30·2 | 0·005 | 1·7 |  | **PB48** | 0·887 | 74·0 | 0·009 | 3·4 |
| **PB3** | 0·737 | 3·7 | 0·008 | 1·9 |  | **PB49** | 0·824 | 17·1 | 0·009 | 2·3 |
| **PB4** | 0·713 | 4·5 | 0·006 | 2·6 |  | **PB50** | 0·687 | 38·8 | 0·007 | 2·2 |
| **PB5** | 0·689 | 3·5 | 0·008 | 2·3 |  | **PB51** | 0·538 | 2·0 | 0·007 | 1·5 |
| **PB6** | 0·639 | 4·2 | 0·005 | 1·7 |  | **PB52** | 0·765 | 13·1 | 0·009 | 1·3 |
| **PB7** | 0·624 | 3·5 | 0·004 | 1·7 |  | **PB53** | 0·654 | 44·3 | 0·005 | 2·5 |
| **PB8** | 0·700 | 38·5 | 0·005 | 2·0 |  | **PB54** | 0·606 | 114·4 | 0·012 | 4·3 |
| **PB9** | 0·661 | 30·2 | 0·007 | 2·2 |  | **PB55** | 0·741 | 114·1 | 0·006 | 2·6 |
| **PB10** | 0·644 | 30·6 | 0·005 | 2·2 |  | **PB56** | 0·825 | 32·7 | 0·007 | 1·8 |
| **PB11** | 0·684 | 30·2 | 0·005 | 2·1 |  | **PB57** | 0·707 | 43·7 | 0·005 | 1·6 |
| **PB12** | 0·704 | 30·0 | 0·007 | 1·7 |  | **PB58** | 0·557 | 1·5 | 0·006 | 1·0 |
| **PB13** | 0·611 | 17·1 | 0·005 | 2·2 |  | **PB59** | 0·515 | 34·2 | 0·003 | 4·1 |
| **PB14** | 0·619 | 93·1 | 0·006 | 3·2 |  | **PB60** | 0·458 | 34·7 | 0·003 | 4·0 |
| **PB15** | 0·586 | 45·1 | 0·005 | 1·8 |  | **PB61** | 0·558 | 18·6 | 0·003 | 1·9 |
| **PB16** | 0·547 | 54·5 | 0·006 | 2·4 |  | **PB62** | 0·558 | 13·6 | 0·003 | 2·5 |
| **PB17** | 0·587 | 3·9 | 0·006 | 1·5 |  | **PB63** | 0·550 | 7·5 | 0·004 | 1·5 |
| **PB18** | 0·577 | 26·1 | 0·005 | 1·4 |  | **PB64** | 0·515 | 15·1 | 0·002 | 2·0 |
| **PB19** | 0·569 | 19·3 | 0·006 | 1·6 |  | **PB65** | 0·515 | 11·1 | 0·002 | 1·1 |
| **PB20** | 0·540 | 7·6 | 0·006 | 2·0 |  | **PB66** | 0·603 | 37·8 | 0·004 | 1·6 |
| **PB21** | 0·527 | 6·5 | 0·006 | 1·6 |  | **PB67** | 0·578 | 5·5 | 0·003 | 0·9 |
| **PB22** | 0·515 | 7·5 | 0·005 | 2·3 |  | **PB68** | 0·634 | 30·3 | 0·003 | 1·7 |
| **PB23** | 0·502 | 6·9 | 0·006 | 2·0 |  | **PB69** | 0·653 | 17·1 | 0·006 | 1·6 |
| **PB24** | 0·515 | 1·5 | 0·009 | 0·9 |  | **PB70** | 0·515 | 25·3 | 0·003 | 2·1 |
| **PB25** | 0·490 | 0·5 | 0·006 | 1·2 |  | **PB71** | 0·884 | 152·4 | 0·010 | 4·5 |
| **PB26** | 0·458 | 4·5 | 0·007 | 4·0 |  | **PB72** | 0·780 | 7·9 | 0·007 | 2·8 |
| **PB27** | 0·782 | 30·7 | 0·008 | 2·1 |  | **PB73** | 0·456 | 19·6 | 0·003 | 3·4 |
| **PB28** | 0·609 | 8·5 | 0·006 | 1·8 |  | **PB74** | 0·597 | 64·8 | 0·009 | 2·3 |
| **PB29** | 0·530 | 11·5 | 0·005 | 1·6 |  | **PB75** | 0·596 | 54·9 | 0·008 | 2·9 |
| **PB30** | 0·637 | 17·6 | 0·008 | 2·6 |  | **PB76** | 0·887 | 127·1 | 0·005 | 3·6 |
| **PB31** | 0·796 | 2·3 | 0·009 | 2·0 |  | **PB77** | 0·884 | 37·3 | 0·008 | 1·0 |
| **PB32** | 0·653 | 4·2 | 0·006 | 2·1 |  | **PB78** | 0·640 | 157·7 | 0·007 | 4·8 |
| **PB33** | 0·600 | 22·7 | 0·005 | 1·8 |  | **PB79** | 0·609 | 97·5 | 0·004 | 2·7 |
| **PB34** | 0·586 | 10·6 | 0·005 | 2·1 |  | **PB80** | 0·729 | 67·4 | 0·003 | 2·2 |
| **PB35** | 0·664 | 91·5 | 0·007 | 2·5 |  | **PB81** | 0·810 | 3·0 | 0·004 | 2·0 |
| **PB36** | 0·568 | 13·6 | 0·005 | 1·6 |  | **PB82** | 0·762 | 192·0 | 0·007 | 5·2 |
| **PB37** | 0·596 | 34·1 | 0·006 | 2·1 |  | **PB83** | 0·579 | 2·6 | 0·004 | 1·0 |
| **PB38** | 0·609 | 44·2 | 0·005 | 2·2 |  | **PB84** | 0·572 | 7·0 | 0·004 | 1·1 |
| **PB39** | 0·607 | 4·0 | 0·006 | 1·7 |  | **PB85** | 0·780 | 13·0 | 0·005 | 1·6 |
| **PB40** | 0·682 | 75·0 | 0·009 | 2·8 |  | **PB86** | 0·611 | 12·1 | 0·006 | 1·4 |
| **PB41** | 0·695 | 17·1 | 0·008 | 2·1 |  | **PB87** | 0·652 | 12·6 | 0·006 | 1·7 |
| **PB42** | 0·740 | 38·8 | 0·008 | 1·4 |  | **PB88** | 0·663 | 20·0 | 0·004 | 1·3 |
| **PB43** | 0·678 | 55·4 | 0·006 | 3·3 |  | **PB89** | 0·625 | 30·6 | 0·005 | 1·4 |
| **PB44** | 0·726 | 8·6 | 0·008 | 1·6 |  | **PB90** | 0·681 | 7·6 | 0·004 | 1·3 |
| **PB45** | 0·666 | 13·6 | 0·005 | 2·1 |  | **PB91** | 0·693 | 28·8 | 0·003 | 2·2 |

RT = retention time; VOC = volatile organic compound

# **Supplementary table 2: Detected volatile organic compounds in fecal samples**

| **VOC** | **1/K0** | **RT** | **1/K0 radius** | **RT radius** |  | **VOC** | **1/K0** | **RT** | **1/K0 radius** | **RT radius** |
| --- | --- | --- | --- | --- | --- | --- | --- | --- | --- | --- |
| **PF0** | 0·859 | 108·2 | 0·005 | 4·6 |  | **PF46** | 0·450 | 63·3 | 0·003 | 5·8 |
| **PF1** | 0·652 | 5·5 | 0·006 | 1·3 |  | **PF47** | 0·547 | 20·1 | 0·005 | 1·8 |
| **PF2** | 0·650 | 0·5 | 0·008 | 0·6 |  | **PF48** | 0·507 | 9·1 | 0·009 | 1·1 |
| **PF3** | 0·548 | 5·0 | 0·007 | 1·1 |  | **PF49** | 0·583 | 28·7 | 0·006 | 1·6 |
| **PF4** | 0·547 | 0·7 | 0·004 | 0·8 |  | **PF50** | 0·771 | 10·6 | 0·004 | 1·2 |
| **PF5** | 0·450 | 4·0 | 0·003 | 2·1 |  | **PF51** | 0·749 | 10·4 | 0·007 | 1·6 |
| **PF6** | 0·506 | 0·5 | 0·003 | 2·2 |  | **PF52** | 0·628 | 27·7 | 0·006 | 1·7 |
| **PF7** | 0·502 | 4·1 | 0·006 | 1·6 |  | **PF53** | 0·583 | 35·6 | 0·007 | 1·6 |
| **PF8** | 0·529 | 5·0 | 0·006 | 0·9 |  | **PF54** | 0·583 | 44·7 | 0·007 | 1·5 |
| **PF9** | 0·528 | 0·5 | 0·006 | 0·7 |  | **PF55** | 0·778 | 18·2 | 0·008 | 1·6 |
| **PF10** | 0·571 | 4·1 | 0·004 | 1·6 |  | **PF56** | 0·684 | 31·6 | 0·008 | 2·0 |
| **PF11** | 0·893 | 109·2 | 0·007 | 5·8 |  | **PF57** | 0·677 | 21·6 | 0·006 | 1·7 |
| **PF12** | 0·853 | 24·7 | 0·005 | 1·7 |  | **PF58** | 0·505 | 14·6 | 0·003 | 2·7 |
| **PF13** | 0·828 | 23·4 | 0·005 | 1·4 |  | **PF59** | 0·505 | 20·6 | 0·003 | 3·1 |
| **PF14** | 0·759 | 55·3 | 0·009 | 2·3 |  | **PF60** | 0·505 | 28·2 | 0·003 | 3·5 |
| **PF15** | 0·726 | 55·5 | 0·005 | 2·5 |  | **PF61** | 0·624 | 17·1 | 0·005 | 1·8 |
| **PF16** | 0·682 | 54·8 | 0·010 | 2·0 |  | **PF62** | 0·610 | 17·6 | 0·005 | 1·7 |
| **PF17** | 0·716 | 32·2 | 0·006 | 1·2 |  | **PF63** | 0·583 | 14·1 | 0·005 | 1·6 |
| **PF18** | 0·700 | 31·7 | 0·005 | 1·3 |  | **PF64** | 0·650 | 2·5 | 0·007 | 1·5 |
| **PF19** | 0·700 | 26·8 | 0·009 | 1·4 |  | **PF65** | 0·670 | 28·2 | 0·003 | 3·8 |
| **PF20** | 0·798 | 11·7 | 0·008 | 1·4 |  | **PF66** | 0·909 | 55·4 | 0·007 | 3·0 |
| **PF21** | 0·785 | 8·6 | 0·006 | 1·2 |  | **PF67** | 0·914 | 24·2 | 0·006 | 2·0 |
| **PF22** | 0·764 | 8·2 | 0·008 | 1·6 |  | **PF68** | 0·649 | 65·4 | 0·005 | 2·0 |
| **PF23** | 0·744 | 7·0 | 0·007 | 1·3 |  | **PF69** | 0·585 | 53·5 | 0·005 | 2·1 |
| **PF24** | 0·720 | 6·6 | 0·008 | 1·7 |  | **PF70** | 0·527 | 18·2 | 0·003 | 3·0 |
| **PF25** | 0·450 | 19·2 | 0·003 | 3·0 |  | **PF71** | 0·848 | 28·2 | 0·005 | 1·3 |
| **PF26** | 0·450 | 29·7 | 0·003 | 3·7 |  | **PF72** | 0·786 | 11·1 | 0·004 | 1·1 |
| **PF27** | 0·669 | 13·7 | 0·008 | 1·5 |  | **PF73** | 0·671 | 10·1 | 0·005 | 1·3 |
| **PF28** | 0·652 | 14·6 | 0·008 | 1·8 |  | **PF74** | 0·528 | 9·1 | 0·005 | 0·8 |
| **PF29** | 0·649 | 23·8 | 0·006 | 1·2 |  | **PF75** | 0·584 | 8·1 | 0·004 | 1·0 |
| **PF30** | 0·563 | 21·7 | 0·006 | 1·5 |  | **PF76** | 0·547 | 15·1 | 0·005 | 1·5 |
| **PF31** | 0·547 | 29·2 | 0·003 | 3·6 |  | **PF77** | 0·645 | 27·7 | 0·006 | 1·3 |
| **PF32** | 0·588 | 19·2 | 0·004 | 1·5 |  | **PF78** | 0·623 | 5·5 | 0·005 | 1·1 |
| **PF33** | 0·594 | 2·0 | 0·005 | 1·5 |  | **PF79** | 0·622 | 0·5 | 0·005 | 1·5 |
| **PF34** | 0·602 | 33·4 | 0·003 | 3·9 |  | **PF80** | 0·554 | 17·5 | 0·004 | 1·2 |
| **PF35** | 0·691 | 7·7 | 0·007 | 1·7 |  | **PF81** | 0·598 | 10·5 | 0·005 | 1·2 |
| **PF36** | 0·586 | 5·8 | 0·009 | 1·0 |  | **PF82** | 0·863 | 32·1 | 0·006 | 1·8 |
| **PF37** | 0·451 | 9·1 | 0·003 | 2·6 |  | **PF83** | 0·769 | 32·1 | 0·006 | 1·3 |
| **PF38** | 0·551 | 9·4 | 0·007 | 1·3 |  | **PF84** | 0·572 | 9·0 | 0·006 | 1·1 |
| **PF39** | 0·617 | 13·7 | 0·007 | 1·4 |  | **PF85** | 0·650 | 45·1 | 0·006 | 3·6 |
| **PF40** | 0·608 | 9·1 | 0·005 | 1·2 |  | **PF86** | 0·639 | 36·6 | 0·007 | 1·6 |
| **PF41** | 0·651 | 9·1 | 0·006 | 1·0 |  | **PF87** | 0·573 | 0·0 | 0·004 | 1·0 |
| **PF42** | 0·640 | 6·6 | 0·006 | 1·1 |  | **PF88** | 0·722 | 0·0 | 0·005 | 1·4 |
| **PF43** | 0·662 | 9·1 | 0·005 | 1·1 |  | **PF89** | 0·720 | 10·2 | 0·007 | 1·0 |
| **PF44** | 0·611 | 6·1 | 0·005 | 1·1 |  | **PF90** | 0·481 | 0·4 | 0·004 | 0·8 |
| **PF45** | 0·717 | 13·2 | 0·007 | 1·6 |  | **PF91** | 0·642 | 9·0 | 0·004 | 1·0 |
| **PF92** | 0·640 | 13·5 | 0·003 | 1·3 |  | **PF140** | 0·672 | 6·5 | 0·005 | 1·0 |
| **PF93** | 0·698 | 22·5 | 0·006 | 1·7 |  | **PF141** | 0·962 | 55·1 | 0·007 | 2·8 |
| **PF94** | 0·692 | 11·0 | 0·006 | 1·3 |  | **PF142** | 0·841 | 18·5 | 0·005 | 2·2 |
| **PF95** | 0·720 | 26·0 | 0·005 | 1·3 |  | **PF143** | 0·825 | 18·5 | 0·007 | 1·8 |
| **PF96** | 0·721 | 22·5 | 0·005 | 1·4 |  | **PF144** | 0·801 | 21·0 | 0·004 | 3·3 |
| **PF97** | 0·645 | 19·5 | 0·004 | 1·3 |  | **PF145** | 0·882 | 54·6 | 0·004 | 3·2 |
| **PF98** | 0·637 | 21·0 | 0·004 | 1·1 |  | **PF146** | 0·959 | 131·1 | 0·005 | 4·4 |
| **PF99** | 0·679 | 43·1 | 0·004 | 1·1 |  | **PF147** | 0·772 | 14·0 | 0·004 | 1·1 |
| **PF100** | 0·547 | 61·9 | 0·003 | 6·0 |  | **PF148** | 0·849 | 18·5 | 0·003 | 1·5 |
| **PF101** | 0·836 | 22·0 | 0·004 | 1·5 |  | **PF149** | 0·819 | 28·5 | 0·005 | 1·7 |
| **PF102** | 0·858 | 19·6 | 0·005 | 1·8 |  | **PF150** | 0·933 | 56·0 | 0·005 | 2·7 |
| **PF103** | 0·877 | 13·5 | 0·004 | 2·0 |  | **PF151** | 0·878 | 75·2 | 0·007 | 2·8 |
| **PF104** | 0·681 | 14·0 | 0·004 | 1·6 |  | **PF152** | 0·941 | 75·0 | 0·006 | 2·6 |
| **PF105** | 0·698 | 16·5 | 0·004 | 1·5 |  | **PF153** | 0·896 | 93·7 | 0·006 | 3·4 |
| **PF106** | 0·617 | 10·1 | 0·003 | 1·2 |  | **PF154** | 0·711 | 131·0 | 0·006 | 6·8 |
| **PF107** | 0·744 | 20·1 | 0·004 | 2·0 |  | **PF155** | 0·642 | 156·8 | 0·006 | 7·6 |
| **PF108** | 0·865 | 12·0 | 0·007 | 2·4 |  | **PF156** | 0·665 | 253·3 | 0·005 | 9·4 |
| **PF109** | 0·845 | 11·5 | 0·007 | 1·3 |  | **PF157** | 0·594 | 80·0 | 0·009 | 2·8 |
| **PF110** | 0·823 | 12·0 | 0·005 | 1·3 |  | **PF158** | 0·583 | 91·5 | 0·005 | 4·1 |
| **PF111** | 0·679 | 48·1 | 0·005 | 1·6 |  | **PF159** | 0·582 | 227·1 | 0·004 | 7·4 |
| **PF112** | 0·721 | 41·1 | 0·003 | 4·5 |  | **PF160** | 0·620 | 49·1 | 0·006 | 1·3 |
| **PF113** | 0·744 | 25·0 | 0·004 | 2·1 |  | **PF161** | 0·621 | 228·7 | 0·006 | 7·5 |
| **PF114** | 0·760 | 24·0 | 0·005 | 1·9 |  | **PF162** | 0·604 | 152·2 | 0·006 | 6·7 |
| **PF115** | 0·548 | 50·1 | 0·003 | 2·4 |  | **PF163** | 0·627 | 106·5 | 0·004 | 4·4 |
| **PF116** | 0·539 | 11·2 | 0·003 | 2·7 |  | **PF164** | 0·580 | 76·0 | 0·005 | 5·5 |
| **PF117** | 0·562 | 9·6 | 0·003 | 2·6 |  | **PF165** | 0·583 | 177·2 | 0·005 | 5·2 |
| **PF118** | 0·694 | 2·0 | 0·003 | 2·1 |  | **PF166** | 0·653 | 109·6 | 0·004 | 5·2 |
| **PF119** | 0·513 | 5·1 | 0·004 | 0·9 |  | **PF167** | 0·546 | 39·3 | 0·003 | 4·6 |
| **PF120** | 0·647 | 58·3 | 0·006 | 2·0 |  | **PF168** | 0·538 | 27·4 | 0·003 | 3·7 |
| **PF121** | 0·681 | 17·7 | 0·005 | 1·9 |  | **PF169** | 0·567 | 38·0 | 0·003 | 2·6 |
| **PF122** | 0·668 | 19·2 | 0·004 | 1·3 |  | **PF170** | 0·573 | 64·0 | 0·004 | 1·8 |
| **PF123** | 0·595 | 25·3 | 0·004 | 1·3 |  | **PF171** | 0·554 | 54·0 | 0·004 | 2·5 |
| **PF124** | 0·571 | 15·0 | 0·003 | 2·8 |  | **PF172** | 0·583 | 24·8 | 0·004 | 1·7 |
| **PF125** | 0·559 | 5·5 | 0·004 | 1·4 |  | **PF173** | 0·529 | 6·5 | 0·003 | 0·9 |
| **PF126** | 0·763 | 0·5 | 0·006 | 1·3 |  | **PF174** | 0·597 | 40·8 | 0·003 | 2·8 |
| **PF127** | 0·554 | 250·4 | 0·007 | 5·9 |  | **PF175** | 0·546 | 82·5 | 0·003 | 6·8 |
| **PF128** | 0·722 | 92·8 | 0·006 | 4·3 |  | **PF176** | 0·505 | 46·2 | 0·003 | 4·5 |
| **PF129** | 0·711 | 51·0 | 0·004 | 2·5 |  | **PF177** | 0·504 | 74·2 | 0·003 | 6·1 |
| **PF130** | 0·866 | 23·5 | 0·004 | 1·7 |  | **PF178** | 0·559 | 32·7 | 0·003 | 3·9 |
| **PF131** | 0·749 | 133·3 | 0·005 | 3·4 |  | **PF179** | 0·534 | 46·0 | 0·003 | 2·1 |
| **PF132** | 0·578 | 106·9 | 0·005 | 4·3 |  | **PF180** | 0·524 | 41·0 | 0·003 | 4·2 |
| **PF133** | 0·713 | 66·7 | 0·005 | 2·8 |  | **PF181** | 0·538 | 18·3 | 0·003 | 2·4 |
| **PF134** | 0·684 | 37·1 | 0·005 | 1·3 |  | **PF182** | 0·525 | 14·0 | 0·002 | 1·7 |
| **PF135** | 0·653 | 53·6 | 0·005 | 1·6 |  | **PF183** | 0·525 | 25·5 | 0·003 | 3·7 |
| **PF136** | 0·816 | 7·6 | 0·005 | 1·3 |  | **PF184** | 0·584 | 49·1 | 0·004 | 1·7 |
| **PF137** | 0·720 | 3·3 | 0·005 | 1·3 |  | **PF185** | 0·504 | 7·0 | 0·004 | 1·0 |
| **PF138** | 0·594 | 13·1 | 0·005 | 0·9 |  | **PF186** | 0·572 | 51·5 | 0·003 | 1·4 |
| **PF139** | 0·631 | 3·4 | 0·005 | 1·0 |  | **PF187** | 0·571 | 41·5 | 0·002 | 1·8 |
| **PF188** | 0·686 | 69·0 | 0·004 | 3·5 |  | **PF200** | 0·706 | 24·3 | 0·003 | 1·2 |
| **PF189** | 0·610 | 28·5 | 0·005 | 1·5 |  | **PF201** | 0·649 | 74·7 | 0·003 | 2·5 |
| **PF190** | 0·680 | 3·5 | 0·003 | 2·3 |  | **PF202** | 0·711 | 75·5 | 0·003 | 3·4 |
| **PF191** | 0·649 | 37·0 | 0·003 | 4·1 |  | **PF203** | 0·719 | 86·3 | 0·003 | 2·6 |
| **PF192** | 0·678 | 65·2 | 0·003 | 6·1 |  | **PF204** | 0·562 | 16·2 | 0·003 | 2·8 |
| **PF193** | 0·699 | 41·5 | 0·003 | 4·7 |  | **PF205** | 0·686 | 24·5 | 0·003 | 2·0 |
| **PF194** | 0·619 | 38·2 | 0·003 | 4·1 |  | **PF206** | 0·717 | 56·5 | 0·003 | 1·8 |
| **PF195** | 0·617 | 20·2 | 0·003 | 3·1 |  | **PF207** | 0·708 | 21·5 | 0·003 | 0·9 |
| **PF196** | 0·654 | 19·7 | 0·003 | 2·0 |  | **PF208** | 0·578 | 18·5 | 0·003 | 3·1 |
| **PF197** | 0·725 | 68·7 | 0·003 | 6·1 |  | **PF209** | 0·708 | 10·5 | 0·003 | 2·6 |
| **PF198** | 0·680 | 51·5 | 0·006 | 0·6 |  | **PF210** | 0·654 | 29·0 | 0·003 | 2·3 |
| **PF199** | 0·565 | 7·2 | 0·001 | 1·0 |  |  |  |  |  |  |

RT = retention time; VOC = volatile organic compound

# **Supplementary table 3: Selected volatile organic compounds by lasso regression**

|  | **Pooled IBS vs HC** | **IBS-D vs HC** | **IBS-C vs HC** | **IBS-M vs HC** | **IBS-D vs IBS-C** | **IBS-C vs IBS-M** | **IBS-D vs IBS-M** |
| --- | --- | --- | --- | --- | --- | --- | --- |
| **Breath** | | | | | | | |
| *VOCs^#^* | *PB11, PB31, PB37, PB57, PB58, PB66, PB70, PB81* | *PB7,* ***PB11****, PB31,* ***PB35****,* ***PB37****,* ***PB41****, PB45,* ***PB57****,* ***PB66****, PB70, PB75, PB78, PB80,* ***PB81****, PB89, PB90* | *PB45,* ***PB61*** | ***PB2****,* ***PB14****,* ***PB21****, PB24, PB29,* ***PB31****, PB32,* ***PB36****,* ***PB37****,* ***PB38****, PB44, PB51, PB54, PB55,* ***PB57****, PB58,* ***PB59****, PB60, PB63,* ***PB66****,* ***PB71****, PB73, PB74,* ***PB75****,* ***PB76****,* ***PB77****, PB78, PB80,* ***PB81****,* ***PB83****, PB88* | *PB0,* ***PB7****,* ***PB12****, PB35, PB56,* ***PB61****, PB63, PB71, PB77,* ***PB89*** | ***PB45*** | ***PB2****, PB5,* ***PB11****, PB12,* ***PB14****,* ***PB15****, PB32,* ***PB33****,* ***PB35****, PB42, PB45, PB55,* ***PB58****, PB63, PB71, PB74,* ***PB82****, PB84, PB88, PB89, PB90* |
| **Feces** | | | | | | | |
| *VOCs^#^* | *PF0, PF51,* ***PF9****, PF42, PF49,* ***PF56****, PF94, PF125,* ***PF170****, PF184* | *PF9, PF51,* ***PF56****,* ***PF71****,* ***PF84****,* ***PF90****,* ***PF92****,* ***PF94****, PF105, PF124, PF127, PF138, PF143,* ***PF152****, PF154,* ***PF170****,* ***PF172****, PF180, PF184* | *PF0,* ***PF1****, PF2, PF9, PF10, PF34, PF37,* ***PF49****,* ***PF56****, PF75, PF107, PF119, PF126, PF134,* ***PF170****, PF172* | *PF8,* ***PF9,*** *PF49,* ***PF56****,* ***PF78****, PF125, PF206* | *PF0, PF1, PF11, PF21, PF34,* ***PF37****, PF41,* ***PF43****, PF49, PF51, PF52, PF55, PF80, PF116,* ***PF119****, PF127, PF129, PF140, PF146,* ***PF152****, PF156, PF159,* ***PF172****, PF199, PF208* | *PF32, PF36,* ***PF40****, PF41, PF61, PF63, PF65, PF81, PF87, PF105, PF117,* ***PF125****, PF126, PF131, PF137, PF143, PF183,* ***PF208*** | ***PF3****,* ***PF32****, PF35,* ***PF36****,* ***PF37****,* ***PF40****, PF44,* ***PF51****,* ***PF53****, PF63,* ***PF64****, PF74,* ***PF77****,* ***PF80****, PF90,* ***PF105****, PF111,* ***PF117****,* ***PF123****, PF125, PF131, PF138, PF166, PF172, PF185, PF206,* ***PF208*** |
| **Breath and feces** | | | | | | | |
| *VOCs^#^* | ***PF0****, PF1,* ***PB2****,* ***PF9****,* ***PB11****,* ***PF30****,* ***PB32****,* ***PF42****,* ***PB37****, PB58,* ***PB81****, PB53,* ***PF56****,* ***PB57****,* ***PB58****, PB59, PB62, PB71,* ***PB73****, PF75, PF79,* ***PF94****,* ***PF124****,* ***PF125****, PF127,* ***PF170*** | ***PB11****, PB15, PB32, PB37,* ***PB57****,* ***PB81****,* ***PF56****,* ***PB62****,* ***PB67****, PB84, PF84,* ***PF90****,* ***PF92****,* ***PF94****, PF127,* ***PF146****, PF152,* ***PF170****,* ***PF172****, PF205* | ***PB0****, PF3, PF9, PF22, PB24, PB53,* ***PF56****,* ***PB61****, PF75, PB79, PB81,* ***PB89****, PF119, PF170* | ***PF9****,* ***PF56****, PF78* | *PB0, PF3,* ***PB12****, PB33, PF41, PF43,* ***PF52****, PF53, PB61, PB77,* ***PB89****,* ***PF119****, PF146, PF152, PF163,* ***PF172*** | ***PB0****, PB12, PF32,* ***PB45****, PB61, PF63, PF125, PF143, PF208* | *PB2, PF3, PB7, PB12, PB15, PB30, PB35, PF36, PF37, PB42, PB46, PF49, PF51, PB55,* ***PF63****,* ***PF64****, PB83, PB87,* ***PB89****, PF111, PF119, PF123, PF125, PF144, PF172* |

C = constipation; CI = confidence interval; D = diarrhea; HC = healthy control; IBS = irritable bowel syndrome; M = mixed; VOC = volatile organic compounds *^#^VOCs selected in >30% of the cross-validation models (VOCs in bold are selected in >80% of models).*
